# Fabrication of Functionalized Carbon Nanotube Buckypaper Electrodes for Application in Glucose Biosensors

**DOI:** 10.3390/bios4040449

**Published:** 2014-11-11

**Authors:** Henry Papa, Melissa Gaillard, Leon Gonzalez, Jhunu Chatterjee

**Affiliations:** 1 High Performance Materials Institute, Florida State University, 2005 Levy Avenue, Tallahassee, FL 32310, USA; E-Mails: hap09@my.fsu.edu (H.P.); mgailla@g.clemson.edu (M.G.); 2 Department of Chemical and Biomedical Engineering, FAMU-FSU College of Engineering, 2525 Pottsdamer Street, Tallahassee, FL 32310, USA; E-Mail: lhg12@my.fsu.edu; 3 Department of Biomedical Engineering, Clemson University, Clemson, SC 29634, USA

**Keywords:** electrochemical biosensor, carbon nanotube, buckypaper, glucose sensor, gold nanoparticles

## Abstract

A highly sensitive glucose detection method was developed using functionalized carbon nanotube buckypaper as a free standing electrode in an electrochemical biosensor. Glucose oxidase was immobilized onto various buckypaper samples in order to oxidize glucose resulting in a measureable current/voltage signal output of the biosensor. Cyclic voltammetry (CV) and amperometry were utilized to determine the sensitivity of these buckypaper electrodes. Sensors of three different types of buckypaper were prepared and compared. These modified buckypaper electrode-based sensors showed much higher sensitivity to glucose compared to other electrochemical glucose sensors.

## 1. Introduction

Functionalized carbon nanotube (CNT) buckypapers (BP) were used in an effort to develop a highly sensitive glucose sensor. It has been reported that a relatively low level of glucose in blood would cause hypoglycemia and also could initiate epileptic seizure or even nerve damage [1]. Recent research reports also suggest that it could trigger a heart attack due to very low sugar levels [2]. Hence, the determination of a low glucose level with high sensitivity was very important. This research described the effectiveness of modified CNT BP electrodes for the detection of glucose at relatively low levels.

Buckypaper is a thin sheet (50–60 micron) of carbon nanotubes (CNTs), which is an advanced nanomaterial with outstanding electrochemical, piezoresistive, and mechanical properties. Thus, CNTs show a broad range of potential applications, such as lithium-ion batteries and solar cells [3]. Furthermore, CNTs are also very desirable for biosensor applications because of their physical and chemical stability, as well as their high electrical conductance and electrochemical properties. Various research groups have reported the functionalization of CNT by various molecules/metal nanoparticles [4,5,6,7] enhancing its electrocatalytic action towards a specific biomolecule. Recent research reported electrochemical sensors, specifically for glucose, based on modified CNTs and gold nanoparticles [8,9]. In this research, free standing and more versatile electrodes based on functionalized buckypaper were fabricated using functionalized multiwall and single wall carbon nanotubes. Recently, a growing interest in utilizing buckypaper in glucose detection was reported [4,5]. Gold nanoparticles in biosensor enhance enzyme immobilization, increase retention and enzyme activity without affecting the biological recognition [8,9,10,11]. AuNPs in size range from 5–50 nm facilitates better electron transfer distance between enzyme and electrode [10,11]. Buckypaper made up of carboxyl functionalized CNTs with incorporation of gold nanoparticles could be an excellent platform to immobilize glucose oxidase. Thus, the use of buckypaper as a free standing electrode (without any support) for highly sensitive glucose detection compared to commercial blood glucose monitoring systems [12] was reported in this research article.

## 2. Experimental Section

### 2.1. Materials and Reagents

MWCNTs, SWCNTs were purchased from SWeNT (Norman, OK, USA) Glucose oxidase (Type X-S from Aspergillus niger), α-D-glucose, chitosan, and horseradish peroxidase (HRP) were purchased from Sigma-Aldrich (St. Louis, MO, USA) and were used as received. Acetic acid obtained from Sigma-Aldrich was diluted to 0.2 M using deionized water. Phosphate buffer solution (PBS) (pH = 7.4) was purchased from Sigma-Aldrich and diluted to 0.1 M and stored at 4 °C. All other reagents, also purchased from Sigma-Aldrich, were of analytical grade. Stock solution of α-D-glucose ranging from 0.5–20 mM concentration were prepared in 0.1 M PBS with a pH of 7.4 ,stirred and stored at 4 °C prior to use.

### 2.2. Experimental Methods

#### 2.2.1. Fabrication of Functionalized Buckypaper

**Figure 1:**
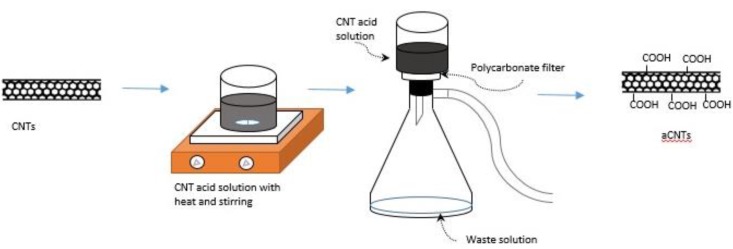
A general schematic for the acid modification of carbon nanotubes (CNTs) is shown.

CNTs were modified by immersing into 1 L of a concentrated sulfuric acid-nitric acid mixture (a 3:1 ratio, *i.e*., 750 mL sulfuric acid to 250 mL nitric acid) for 8 h and stirred at 70 °C according to the conventional procedure [7]. After the acid modification, the dispersion of CNTs was vacuum filtered and washed 3 times using 1 L DI water each time, or until the washed solution reached a pH value of 7. The filtered acid-modified CNTs (a MWCNT or a SWCNT) were removed from the filter paper and placed into an aluminum pan drying in a vacuum oven at 60 °C overnight. For buckypaper samples, carboxyl functionalized CNTs (either one type or mixture of both type CNTs) were dispersed into dimethyl formamide (DMF) using probe sonication for 8 min. This dispersion was mixed with prepared 0.01% of gold nanoparticles which were prepared following a citrate reduction [9] of HAuCl_4_·3H_2_O for a 2-h bath sonication. The gold solution was prepared by mixing 60 mg of HAuCl_4_·3H_2_O with 600 mL DI water. This gold solution was then placed on a hot plate at 157 °C, allowing for evaporation. At the end of the evaporation, a sodium citrate solution of 1.75 g sodium citrate and 120 mL DI water was then added. This solution was cooled inside a dark color bottle). The dispersion with gold was further filtered resulting in the formation of the buckypapers (BP1, BP2 and BP3). The amount of gold calculated was about 30 mg (*i.e.*, 25% by weight of CNT) in 120 mg of CNT. Table 1 lists the basic composition of these buckypaper samples. The glucose oxidase (GOx) enzyme, 20 µL of 5 mg/mL GOx, 10 µL of 2 mg/mL HRP, and 10 µL of 2 mg/mL chitosan were mixed into a solution for the immobilization of the enzyme. This solution was cast onto a small sample (4 mm × 5 mm) of BP-1, BP-2 and BP-3. A schematic diagram of this fabrication process in the functionalization of the electrode is shown in Figure 1.

**Table 1:** Buckypaper sample description and composition of electrodes for glucose oxidation.

BP Sample	Composition and processing	GOx Immobilization
BP-1	60 mg aMWCNT + 60 mg aSWCNT DMF solution (sonicated together with CNT) AuNP (bath sonicated)	10 μL of 2 mg/mL HRP 10 μL of 2 mg/mL chitosan 20 μL of 2 mg/mL GOx
BP-2	120 mg aSWCNT DMF solution (sonicated together with CNT) AuNP (bath sonicated)	10 μL of 2 mg/mL HRP 10 μL of 2 mg/mL chitosan 20 μL of 2 mg/mL GOx
BP-3	120 mg aMWCNT DMF solution (sonicated together with CNT) AuNP (bath sonicated)	10 μL of 2 mg/mL HRP 10 μL of 2 mg/mL chitosan 20 μL of 2 mg/mL GOx

#### 2.2.2. Characterization of Buckypaper

##### Fourier Transform Infrared Spectroscopy (FTIR) for Acid Modified Carbon Nanotubes

**Figure 2:**
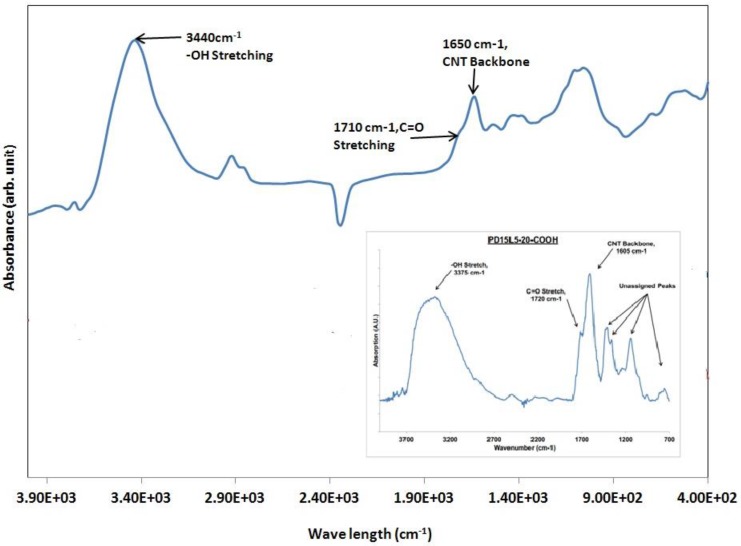
FTIR results for the acid-modified carbon nanotube. The inset in this figure shows FTIR spectra for commercial carboxyl modified multiwalled nanotubes (obtained from Nanolab, Waltham, MA, USA).

Thermo Nicolet 6700 Fourier transform infrared (FT-IR) Spectrometer was used to confirm and assess the modifications to CNTs. Individual palettes for 8-h multi-wall, 8-h single-wall, and 24-h single-wall acid-modified nanotubes were created by mixing 1 mg of a CNT to 99 mg of KBr. The mixture was then physically ground until the nanotubes were evenly distributed throughout the KBr, without any noticeable aggregates. The ground powder of KBr and CNTs were placed under 3 metric tons of pressure for 5 s. The palette was retrieved and loaded into the FT-IR for the spectra measurement. The experimental results are shown in Figure 2.

##### Electron Microscopy and Electrical Conductivity

Both scanning and transmission electron microscopy were performed for the buckypaper samples. A JEOL, JSM-7401F USA, Inc scanning electron microscope was used for electron microscopy technique.

The electrical conductivity as measured by the 4-probe method using a 4-probe station (Jandel,UK) attached with nanovoltmeter and AC/DC current source (Keithley, Fotronic Corporation, MA, USA). A 20 mm × 20 mm film of each buckypaper sample was used to measure the conductivity.

##### Electrochemical Characterization and Sensor Evaluation

Electrochemical experiments were carried out in a regular, standard glass cell (part number, AKCELL1, Pine Instruments) with a three electrode system using a Ag/AgCl reference electrode, platinum counter electrode (platinum mesh, 45 mesh woven from 0.198 mm dia pt. wire fitted with platinum wire, thickness, 0.404 mm) and the buckypaper as working electrode. A small piece of buckypaper sample (4 mm × 5 mm) was placed in a platinum mesh (same as the counter electrode) which acted as a working electrode. A Potentiostat (Versastat 3, Princeton Applied Research, NJ, USA) equipped with Versa Studio software was used to carry out electrochemical experiments. Cyclic voltametry experiments were carried out at different voltage ranges −1 V to +1 V *versus* Ag/AgCl reference electrode, and a scan rate of 50 mV/s.

The cyclic voltammograms of each buckypaper electrode before and after immobilization with enzymes were taken in a blank PBS and in glucose-PBS solutions. The redox potentials indicated that the oxidation and reduction process and the corresponding peak currents of the glucose reactions. As the area of each buckypaper sample was the same (20 mm^2^), the current values were not divided by the area in the CV plots, but this was taken into consideration later while calculating the sensitivity of each biosensor (in Section 3.3). Amperometric analyses were performed with varying concentrations of glucose and time. The corresponding current was measured after certain intervals (100 s), adding a fixed volume (100 microliter, 500 microliter) of glucose solution (1 mM, 20 mM).

## 3. Results and Discussion

### 3.1. Structure and Morphology for Buckypaper Electrode

The FTIR results for the acid-modified carbon nanotube are shown in Figure 2. The inset in this figure shows FTIR spectra for commercial carboxyl modified multiwalled nanotubes (obtained from Nanolab, Waltham, MA, USA) In both cases an –OH stretch can be observed around 3375 cm^−1^. A C=O stretch could be seen for each sample around 1720 cm^−1^. The CNT backbone was shown for each around 1605 cm^−1^, and the unassigned peaks in each case are similar in each spectrum. These results suggested that the CNTs were successfully acid-modified.

**Figure 3:**
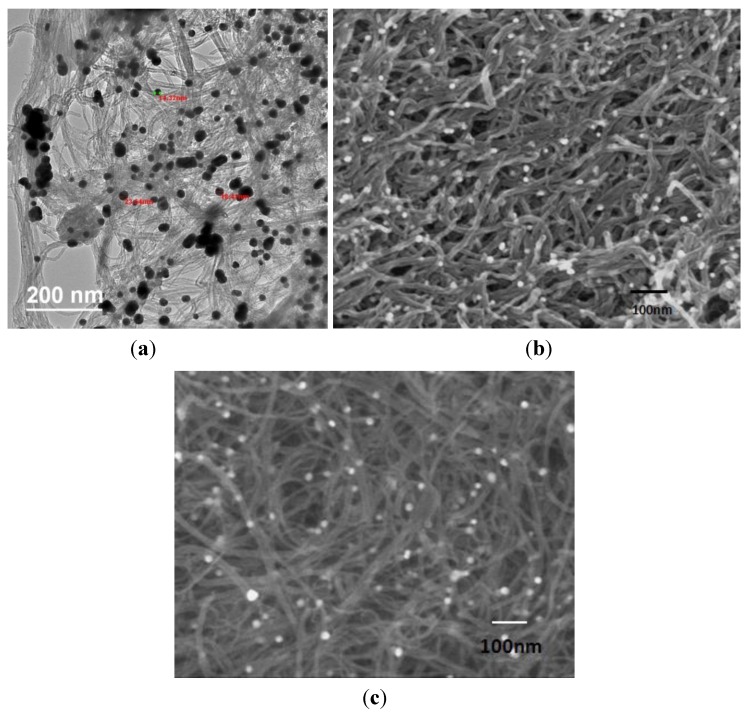
(**a**) Transmission electron microscopy image for buckypaper (BP) 1, (**b**) Scanning electron microscopy image for BP 2 and (**c**) scanning electron microscopy for BP 3.

Morphology for both acid and gold modified buckypapers (BP1, BP2, and BP3) are shown in Figure 3a–c, respectively. Figure 3a is the transmission electron micrograph for BP1 and Figure 3b,c are the scanning electron micrographs. These buckypapers showed the entanglements of carbon nanotubes. Gold nanoparticles are adhered to the inside and outside walls of the CNTs. No apparent significant difference existed between the morphology of SWCNT or MWCNT based buckypapers. Figure 3a shows a mixture of nanotubes with different diameters, possibly due to the presence of both single walled and multiwallled nanotubes.

The electrical conductivities measured by the 4-probe method were 55 s/cm, 160 s/cm and 230 s/cm for BP1, BP2 and BP3 samples, respectively.

### 3.2. Electrochemical Properties for Buckypaper Biosensors

**Figure 4:**
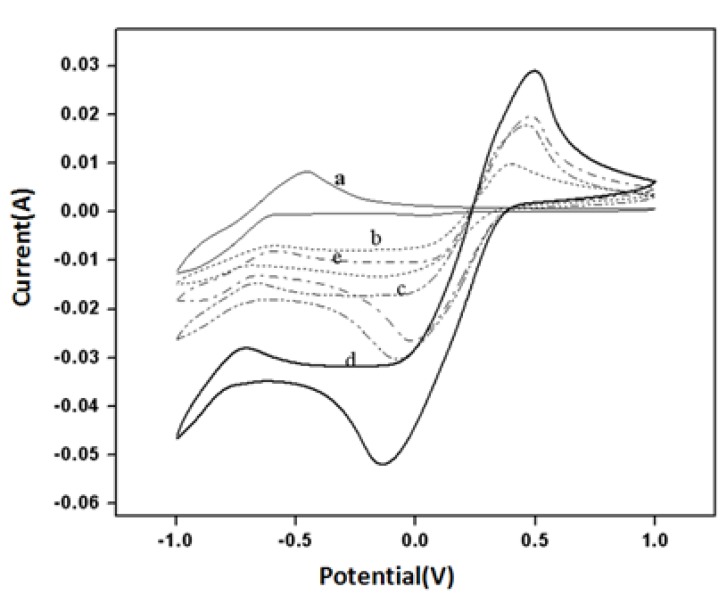
(**a**) pure BP in PBS; (**b**) Pure BP in K_3_Fe(CN)_6_; (**c**) aMWCNT BP with in K_3_Fe(CN)_6_; (**d**) BP1 (aMWCNT with gold) in K_3_Fe(CN)_6_; (**e**) BP1 in K_3_Fe(CN)_6_ with HRP-GOx.

Figure 4 shows the cyclic voltammograms for a pure MWCNT BP without any gold nanoparticles in 0.1 M PBS (curve a), in 0.1 M K_3_Fe(CN)_6_ (curve b) in 0.1 M PBS, for acid modified MWCNT (aMWCNT) in 0.1 M K_3_Fe(CN)_6_ in 0.1 M in (curve c) and finally BP3 in 0.1 M K_3_Fe(CN)_6_ in 0.1 M PBS (curve d) and + BP3/GOx-HRP in 0.1 M K_3_Fe(CN)_6_ + 0.1 M PBS (curve d) were recorded at a voltage scan rate of 50 mV/s. Oxidation and reduction peak currents due to Fe^+3^/Fe^+2^ redox couple were clearly observed even with pure MWCNT BP. However, the oxidation peak current for all acid-modified MWCNT (aMWCNT) electrodes has been increased (curve b, c and d). The increase of oxidation peak current in BP1 was about 60% compared to acid-modified MWCNT and it was about 95% compared to that for pure BP samples. These results showed that acid modification and presence of gold nanoparticles caused a significant enhancement of the electrical signal.

**Figure 5:**
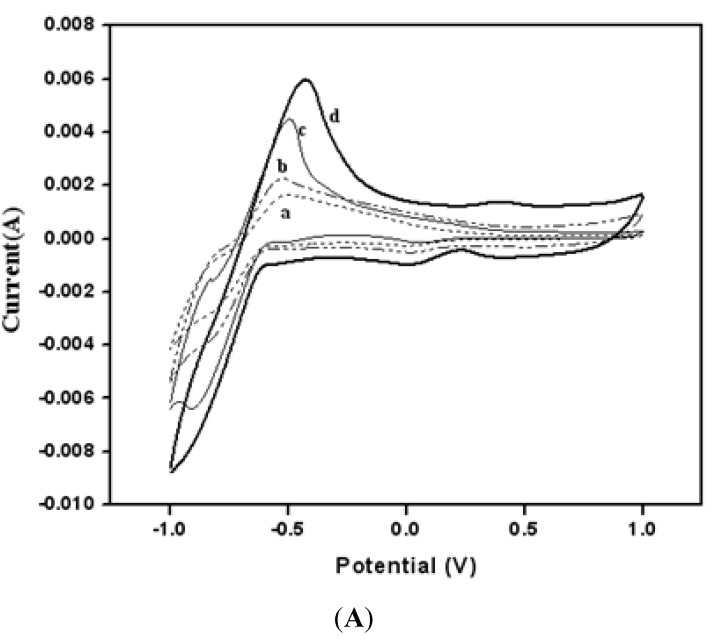

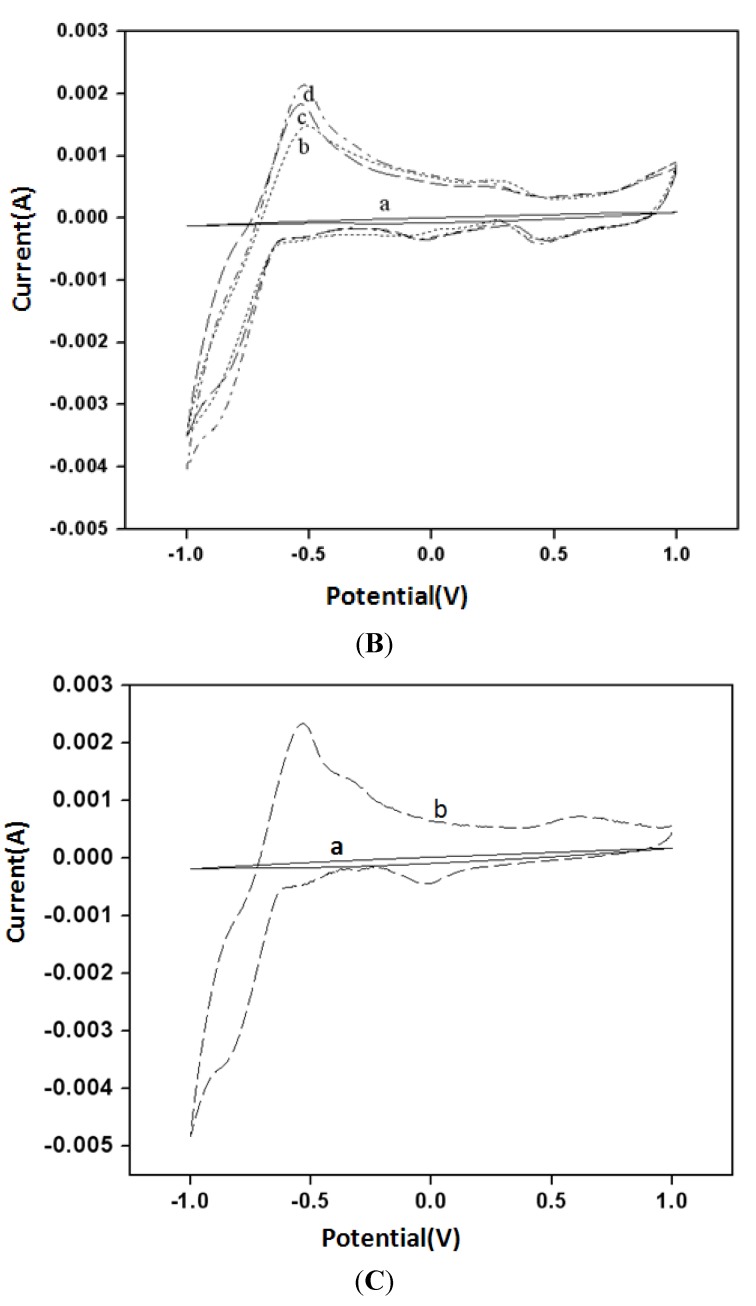
(**A**) Cyclic voltammograms for (a) 0.01 M PBS buffer, (b) 1 mM glucose, (c) 5 mM glucose and (d) 10 mM Glucose solution using BP 1 as working electrode; (**B**) Cyclic voltammograms for (a) 0.01 M PBS buffer, (b) 5 mM glucose, (c) 10 mM glucose, (d) 20 mM glucose using BP 2 as working electrode; (**C**) Cyclic voltammograms for (a) 0.01 M PBS buffer, (b) 20 mM glucose using BP 3 as a working electrode.

Cyclic voltammetry results for all buckypaper samples (BP1, BP2 and BP3) are shown in Figure 5A–C, respectively. The faradic current increased with increase in glucose concentration. These results were in accordance with the common ranges of the glucose oxidation peak (0.33–0.5 V) [11,13,14]. The peak currents, which were more prominent for BP1(combination of single and multiwalled CNT and gold nanoparticles) and BP2 (combination of SWCNT and AuNP) showed that a better oxidation response to glucose was achieved for single walled CNTs with gold particles compared to the buckypaper made with only MWCNT and gold nanoparticles.

### 3.3. Amperometry and Glucose Biosensor Sensitivity

Amperometric studies were performed for the three types of buckypapers by addition of a specific volume of a pure glucose solution.

**Figure 6:**
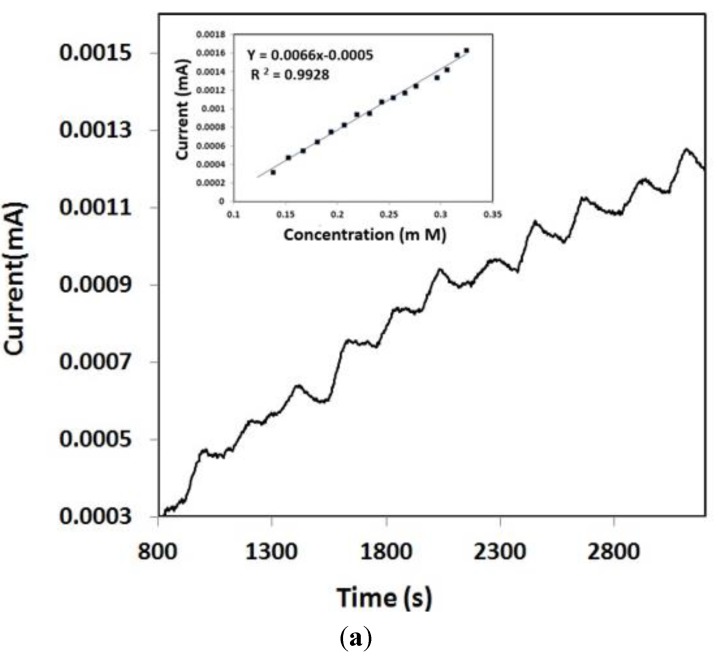

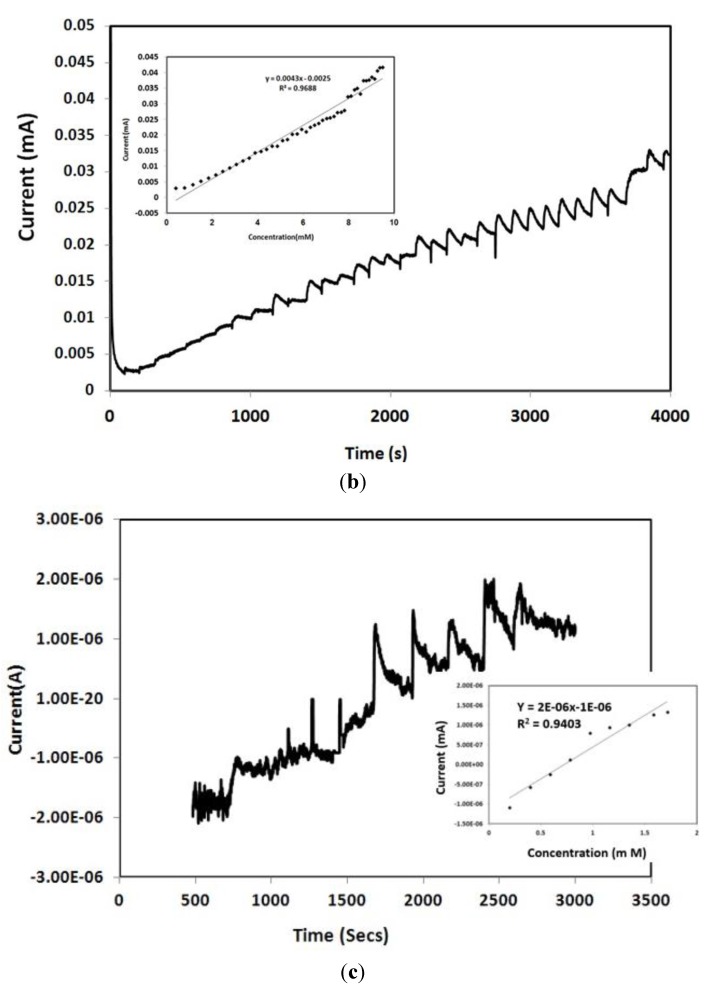
(**a**) Amperometric response and calibration curve using BP1 electrode upon addition of 100 µL of 1 mM glucose after every 100 s; (**b**) Amperometric response and calibration curve using BP2 electrode upon addition of 100 µL of 20 mM glucose after every 100 s; (**c**) Amperometric response and calibration curve using BP2 electrode upon addition of 500 µL of 20 mM solution glucose solution was done after every 100 s.

For BP 1, successive addition of 100 µL of 1 mM glucose solution was performed. The amperometry results are shown in Figure 6a along with the corresponding calibration plot (inset). The sensitivity and the limit of detection obtained were 18 µA·cm^−2^/mM and LOD 0.025 mM, respectively.

Figure 6b represents amperometry results and the calibration plot derived from it using BP 2 electrode. Successive addition of 100 µL of 20 mM glucose after every 100 s was performed. A steady current reached within a relatively short time and the amperometric measurements were obtained. The inset plot in Figure 6b showed the calibration plot for glucose concentration over the range of 0–10 mM. (Normal physiological glucose concentration range is from 3–7 mM). The sensitivity and the detection limits were calculated as 21.5 µA·cm^−2^/mM and 0.0215 mM. For BP 3 (Figure 6c) addition of 500 µL 20 mM solution glucose solution was done after every 100 s and the sensitivity and the limit of detection were 10 µA·cm^−2^/mM and 2.65 mM, respectively. The amount of current decreased in Figure 6c compared to Figure 6b and Figure 6a because of the difference in electrochemical sensitivity of the buckypaper samples (BP1, BP2 and BP3).

The sensitivity and limit of detection was at the highest point for the buckypaper electrode fabricated with acid modified SWCNT (BP 2) and gold nanoparticles compared to the other BP electrodes (BP1 and BP 3).

### 3.4. Selectivity of the Biosensor

**Figure 7:**
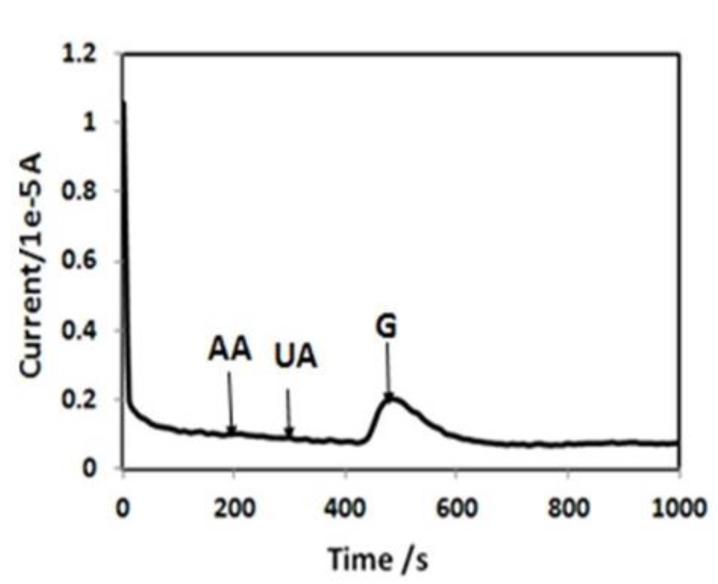
Chronoamperometric response of BP2 electrode biosensor to glucose, ascorbic acid (AA) and uric acid (UA) at a potential of 0.5 V (physiological level of AA and UA were added (0.1 mM for AA, 0.1 mM for UA) at the 100th and 200th second, respectively, and subsequently 1 mM of glucose was added).

To investigate the selectivity of the CNT BP-based biosensor, its response towards common interfering species, such as ascorbic acid (AA) and uric acid (UA), was investigated. As shown in Figure 7 (chronoamperometry at 0.5 V), for BP2, there is no significant current response upon the addition of interfering molecules at their normal physiological level (0.1 mM for AA, 0.1 mM for UA) in the 100th and 200th second, respectively. In contrast, a strong current response was observed by addition of 1 mM glucose in the presence of AA and UA. Thus, specific quantification of glucose is possible with this acid when modified by BP/GOx electrode.

## 4. Conclusions

Free standing buckypaper electrodes were successfully fabricated for glucose detection. Attaching carboxyl groups and gold nanoparticles on carbon nanotubes has significantly enhanced the electrochemical sensitivity of the buckypapers. These buckypaper electrodes can be used to fabricate glucose sensing strips in a more cost-effective way than the currently available strips and will provide more affordable glucose monitoring. Higher sensitivity towards glucose detection was obtained with these fabricated electrodes.
